# Editorial: Sex steroid metabolism and obesity: insights from animal studies

**DOI:** 10.3389/fendo.2026.1940053

**Published:** 2026-07-30

**Authors:** Laura J. den Hartigh, Mita Varghese

**Affiliations:** 1 University of Washington Medicine Diabetes Institute, University of Washington, Seattle, WA, United States; 2 Department of Pediatrics, Michigan Medicine, University of Michigan, Ann Arbor, MI, United States

**Keywords:** adipose tissue, aging, estrogen, Hypothalamic-pituitary-ovarian (HPO) axis, inflammation, insulin resistance, nuclear factor erythroid 2-related factor 2 (Nrf2), Polycystic ovary syndrome (PCOS)

Obesity rates are rapidly increasing globally, with marked differences in fat distribution often reported between men and women that contribute to the risk of obesity-associated complications such as diabetes and cardiovascular disease. Much research to date has underscored the complex relationship between sex hormones, such as estrogen and testosterone, and the regulation of body weight, fat distribution, and metabolism (1, 2). Significant gaps remain in our understanding of how sex hormones modulate energy balance and influence the development, progression, and complication risks associated with obesity. This Research Topic sought to address mechanistic gaps between sex hormones and adipose tissue dysfunction associated with diet-induced obesity, with a primary focus on animal models.

An introduction to the notion that adipose tissue is an important source of estrogens, particularly in aged women, was provided by Lee and Den Hartigh. This comprehensive review provides evidence that estrogens and testosterone not only directly impact adipose tissue metabolism, but that adipose-derived estrogens, particularly estrone (E1), may contribute to the increased risk for metabolic disease observed in post-menopausal women as well as obesity. White adipose tissue (WAT) expresses high levels of aromatase, which can convert circulating androgens into estrogens, a process that tends to favor estrone conversion in WAT (3). Strong correlations between circulating E1 levels and body weight, BMI, waist circumference, and insulin resistance have been reported in both men and women, with similar associations found in aged women. By contrast, circulating 17β-estradiol (E2) exhibits a strong inverse association with obesity measures and insulin resistance. Similar associations have been found between adipose tissue E1 content and metabolic dysregulation, and adipose E2 content has also been reported to associate with excess adiposity, in stark contrast to circulating E2. Evidence from animal models suggests that estrogens exert differential effects on visceral (i.e. gonadal) and subcutaneous WAT (4), providing a potential mechanism for more beneficial adiposity profiles in pre-menopausal women. The authors conclude that an increased understanding of the interplay between the human WAT hormonal milieu and metabolic health would inform the treatment of metabolic dysfunction in diverse populations.

Estrogen has been shown to be protective against adipose tissue inflammation, while testosterone may impair adipose metabolic function (5–7). Whether estrogen and testosterone directly contribute to these sex differences in obese adipose tissue dysfunction have been demonstrated through gonadectomy and androgen or estrogen receptor perturbation studies (5, 7–10). Elegant work by Skibniewska et al. supports major differences in inflammation and glucose metabolism early in the development of metabolic impairment between male and female mice with short-term diet-induced obesity. Notably, male mice appeared to have more expandable gonadal WAT than females, with earlier markers of inflammation, reduced adipogenesis genes, and elevated insulin levels than females, suggesting a potential role for estrogens in restricting visceral adipose expansion. Further, insulin resistance preceded adipose tissue inflammation in males, suggesting that impaired insulin signaling is an important driver of adipose tissue inflammation early in the development of obesity in males. The authors’ work highlights important sex differences in adipocyte hypertrophy and inflammation that may serve as earlier and more accurate predictors of metabolic dysfunction other than BMI.

A comprehensive overview of adipose-androgen interactions, which is considered crucial for developing precision treatments for polycystic ovary syndrome (PCOS), is presented in a review by Ni et al. It is increasingly recognized that a bidirectional feedback loop involving adipose tissue and hyperandrogenism contributes to PCOS (11). PCOS is more prevalent in people with overweight or obesity, in which dysfunctional WAT promotes insulin resistance, exacerbating a chronic low-grade inflammation that drives excessive ovarian androgen production. This hyperandrogenism worsens WAT dysfunction and insulin resistance by promoting central obesity, resulting in a feedforward loop. This model provides a cohesive pathophysiological framework that explains the intricate link between metabolic and reproductive disturbances in PCOS that leads to reproductive dysfunction and makes a compelling case for targeting WAT function for long-term management of this complex condition.

A review by Chen et al. discusses the complex relationships between adipose tissue and endocrine hormones, such as leptin, insulin, and sex hormones that must be considered during obesity therapy. This manuscript takes a deep dive into mechanisms linking obesity with hypothalamic-pituitary-ovarian (HPO) axis dysfunction. There is a thorough examination of the role of neuronal pathways involved, brain insulin and leptin resistance, and inflammation in the brain. The mechanisms by which obesity disrupts ovarian function, including oocyte meiosis and maturation, oocyte mitochondrial damage, and the general ovarian microenvironment and aging are thoroughly discussed. Additional considerations for a potential role of the gut microbiota in disrupting sex hormone metabolism involving the brain and ovaries is also an important topic. Finally, how the HPO axis should be considered in behavioral, therapeutic, and surgical obesity treatment is explored.

Finally, work by Zhao et al. showcases the importance of adipocyte nuclear factor erythroid 2-related factor 2 (Nrf2) in driving obesity-associated adipose tissue inflammation. Nrf2 is a key regulator of the cellular response to oxidative stress in adipose tissue that controls the expression of antioxidant and detoxification enzymes required to quench excess reactive oxygen species (ROS). Nrf2 has previously been shown to be important for adipogenesis, regulating adipogenic factors such as PPARγ (12). In this study, mice with adipocyte-specific deletion of Nrf2 were protected from high fat diet-induced obesity, glucose dysregulation, and adipose tissue and systemic inflammation through a mechanism involving the cGAS-STING pathway. These findings contradict previously reported protective roles for Nrf2 in inflammation and diet-induced obesity (13), which the authors speculate suggests an important context dependency shaped by Nrf2 source tissue and metabolic microenvironment.

In sum, this Research Topic encompasses comprehensive reviews of estrogen-adipose biology related to metabolic disease and aging; the intersection of hyperandrogenism and adiposity related to PCOS; and important links between the HPO axis and adipokines that should be considered during anti-obesity therapy. Original work also showcases the critical importance of sex hormones in driving metabolic disease progression in mice, with insulin resistance preceding adipose tissue inflammation in males. Finally, a crucial role for adipocyte Nrf2 in the metabolic complications of obesity was described in mice.
